# Minor Sphincter Sparing Surgery for Successful Closure of Perianal Fistulas in Patients with Crohn’s Disease

**DOI:** 10.3390/jcm10204721

**Published:** 2021-10-14

**Authors:** Jennifer Merten, Ann-Kathrin Eichelmann, Rudolf Mennigen, Isabelle Flammang, Andreas Pascher, Emile Rijcken

**Affiliations:** 1 Department of General, Visceral and Transplantation Surgery, University Hospital Muenster, Albert-Schweitzer-Campus 1, Building W1, 48149 Muenster, Germany; Ann-Kathrin.Eichelmann@ukmuenster.de (A.-K.E.); Isabelle.Flammang@ukmuenster.de (I.F.); Andreas.Pascher@ukmuenster.de (A.P.); 2 MVZ Portal 10, Albersloher Weg 10, 48155 Muenster, Germany; mennigen@mvz-portal10.de (R.M.)

**Keywords:** Crohn’s disease, perianal fistula, complex fistula, seton drainage, anal fistula plug, OTSC

## Abstract

The purpose of this study is to demonstrate that repetitive minor surgical procedures allow for a high rate of permanent closure of perianal fistulas in patients with Crohn’s disease (CD). Patients with perianal fistulizing CD (PFCD) who underwent perianal surgery at the University Hospital of Muenster between 2003 and 2018 were assessed for fistula characteristics and surgical procedures. We included 45 patients (m:f = 28:17) with a mean age of 27 years at first fistula appearance. Of these, 49% suffered from a complex fistula. An average of 4.2 (1–14) procedures were performed, abscess incisions and fistula seton drainages included. Draining setons were left in place for 5 (1–54) months, until fistula closure. Final surgical techniques were fistulotomy (31.1%), seton removal with sustained biological therapy (26.7%), Anal Fistula Plug (AFP) (17.8%), Over-The Scope-Clip proctology (OTSC) (11.1%), and mucosa advancement flap (4.4%). In 8.9% of cases, the seton was kept as permanent therapy. The time from first to last surgery was 18 (0–182) months and the median follow-up time after the last surgery was 90 (15–200) months. The recurrence rate was 15.5% after 45 (17–111) months. Recurrent fistulas healed after another 1.86 (1–2) surgical re-interventions. The final success rate was 80%. Despite biological treatment, PFCD management remains challenging. However, by repeating minor surgical interventions over a prolonged period of time, high permanent healing rates can be achieved.

## 1. Introduction

Perianal fistulas are a frequent problem in CD patients: population-based studies demonstrate that approximately every fifth CD patient suffers from perianal fistulas and that this risk is even higher in case of colorectal manifestation [1,2]. An association between the length of the CD-course and increasing incidence of PFCD has been described: while one year after initial CD diagnosis, only 12% of the patients showed PFCD, roughly every fourth patient (26%) had a history of perianal fistula after 20 years of disease [3].

PFCD is mostly present in patients under 30 years old and associated with symptoms, such as perianal pain and purulent or fecal discharge. In case of chronic inflammation, the destruction of the anal sphincter and perianal scars can lead to fecal incontinence [1]. These symptoms, especially in combination with further CD-associated complaints, such as abdominal pain, diarrhea, and weight loss, may cause physical exhaustion, psychological distress, a reduction in sexual function and, therefore, a significant impairment of the quality of life in patients.

The American Gastroenterological Association (AGA) [4] classifies perianal fistulas as either simple or complex. Complex fistulas are characterized by a high course towards the sphincter, more than one external opening, and clinical symptoms of a perianal abscess. Complex fistulas in CD can be related to anorectal strictures or endoscopic signs of rectal disease activity. PFCD requires a multidisciplinary approach, consisting of medical therapy (biologicals, antibiotics) and in most cases surgical interventions to achieve symptom relief or fistula healing [5]. In addition to the medical treatment, surgery represents a key component in the management of PFCD. First, a conditioning seton drainage can be placed, so as to prevent further abscess formation and/or sepsis. Second, procedures such as fistulotomy for simple fistulas and several sphincter sparing surgical treatments for complex fistulas are available. However, the presence of active proctitis precludes a surgical closing approach [6].

Because of high fistula recurrence rates of up to 43% in patients with ileocolic CD as well as high re-intervention rates of 27% in patients with simple and 40% in patients with complex fistulas, treatment of PFCD is challenging [1,7]. In PFCD cohort studies patients need about six surgical procedures (1–23) for complex and three (1–12) for simple perianal fistulas until final fistula closure [8]. Despite the multidisciplinary approach, therapy refractory cases still require major surgery like temporary or final fecal diversion or proctectomy [3,9].

Treatment of CD patients may be reserved to specialized centers, but it is important to note that CD often manifests itself initially by a perianal abscess based on a perianal fistula. Hence, many surgeons encounter PFCD in daily practice. The present study examines UKM PFCD patient histories over the last 15 years with particular focus on minor, anal sphincter sparing surgery, and demonstrates that successful fistula closure in CD can also be achieved by a combination of different approaches.

## 2. Materials and Methods

### 2.1. Study Population

**Figure 1:**
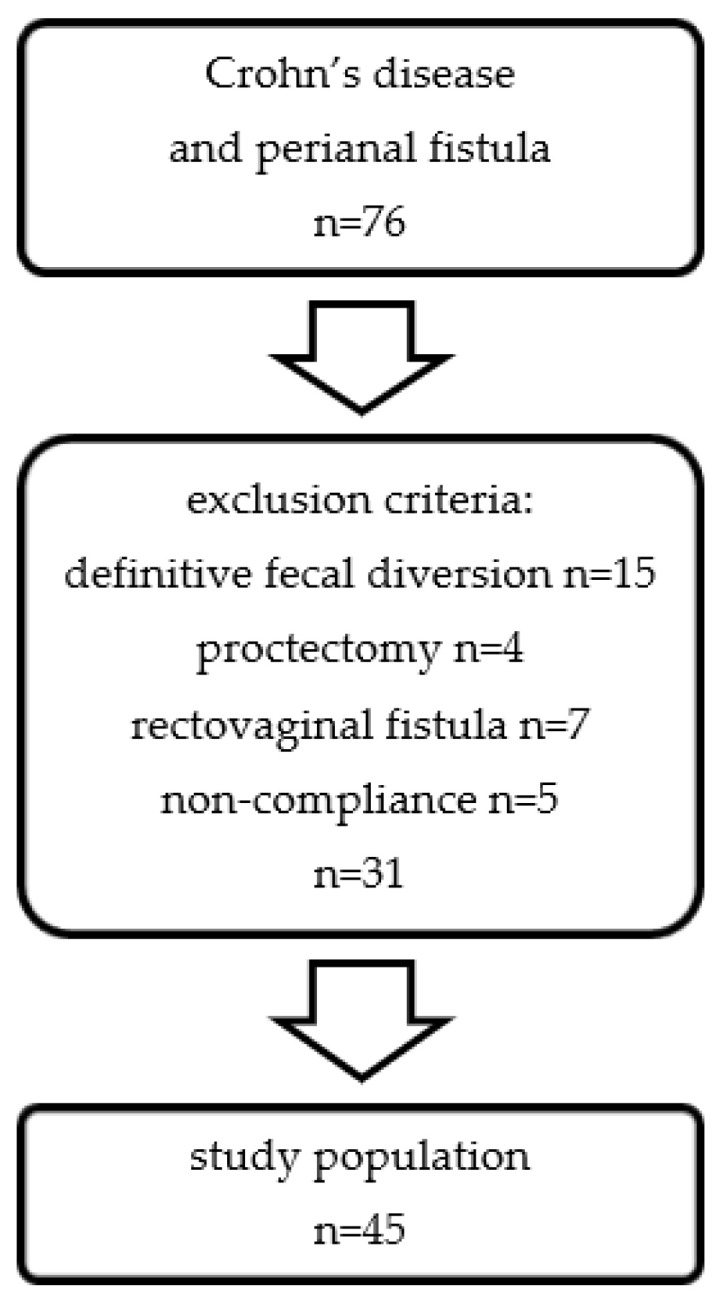
Flow diagram of study cohort in patients with CD and perianal fistula.

All CD patients who underwent perianal fistula surgery between July 2003 and July 2018 at the Department of General, Visceral and Transplantation Surgery of the University Hospital of Muenster, Germany, were retrospectively included. Excluded were patients with final fecal diversion, proctectomy, rectovaginal fistula, or non-compliance (Figure 1). The study was performed in accordance with the ethical standards of the institutional and national research committee (Ethikkommission Muenster, 2018-383-f-S) as well as the Helsinki declaration and its later amendments. Informed consent was obtained from all patients.

### 2.2. Perianal Fistula Treatment

All CD patients presenting with symptoms such as progressive anal or perianal pain, signs of perianal infection or visible external opening of fistula and fistula secretion, underwent a clinical examination, including inspection and pre- or intraoperative digital rectal examination, proctoscopy, and anorectal ultrasound (ERUS), to locate the fistula tract to the anal sphincter. All patients underwent magnet resonance imaging (MRI) before fistula surgery. The presence of an abscess associated with severe pain, fever, or elevated serum inflammatory parameters prompted immediate surgical intervention. Non-irritable perianal fistulas underwent elective surgery. Preoperatively, an enema was administered.

### 2.3. Surgical Management

All patients received a prophylactic single shot intravenous antibiotic injection of cefuroxime and metronidazole 30 min before surgery. They underwent a proctoscopy in lithotomy position under general anesthesia. Surgical management for perianal abscess incision included a fusiform excision to drain the abscess. In this acute inflammatory situation, the fistula tract was carefully probed. To control inflammation and to condition the fistula tract, a seton drainage was placed.

Fistula closure surgery was performed approximately 5 months after seton placement. Preoperatively it was ensured that the conditioning of the fistula tract had been successful and that there was no perianal infection or severe proctitis. Superficial fistulas were excised, and complex fistulas underwent several surgical interventions by senior surgeons so as to close the internal fistula opening: Depending on the course of the fistula tract and its proximity to the anal sphincter, a mucosa submucosa flap, an application of AFP combined with a mucosa advancement flap or an OTSC procedure was performed. Before closure, the external fistula opening was excised and a debridement of the fistula tract was performed.

### 2.4. Postoperative Standard Care

Postoperative standard care consisted of immediate food intake, administration of pain medication, and laxatives to ensure soft stools. One week after discharge, all patients were examined in our outpatient department. Further presentations were arranged on an individual basis, depending on the implemented surgical procedure and the healing progress. Some patients received treatment with biologicals simultaneously. Provided there were no signs of proctitis or inflammation, in some of these cases the seton drainage was removed in week eight after its placement, at the same time as the last administration of biologicals.

### 2.5. Data Collection

Data were collected from the patients’ electronic medical records. This included patient characteristics, such as age at initial fistula appearance, sex, body mass index (BMI), American Society of Anesthesiologists (ASA) severity score, smoking history, medication history, Montreal classification [10], and surgical history. In addition, the initial diagnosis of CD, the first and last fistula surgery, and the last follow-up after the beginning of data collection in July 2020 were recorded. The description, activity, and number of perianal fistulas included preoperative examination and special imaging. Fistulas were classified according to Parks [11] and AGA classifications [4]. Localization of the external fistula opening was described as ventral (10°°–2°°), dorsal (4°°–8°°), and horizontal (9°° + 3°°) in lithotomy position.

An important aspect of the study was a detailed assessment of surgical interventions. This included the various procedures used to close perianal fistulas, perianal disease activity index (PDAI) [12], leukocytosis, the technique used for the last fistula surgery, the number of attempts to close the internal ostium, postoperative complications, and the presence of fecal diversion.

### 2.6. Fistula Healing, Persistence, and Recurrence

Besides fistula healing, which was defined as a complete clinical closure of internal and external opening without signs of fistula activity, i.e., fistula secretion, pain, or inflammatory signs, we distinguished between persistence and recurrence of the perianal fistula. Imaging was not specifically used for final evaluation of fistula healing. Persistence despite technical closure was defined as fistula secretion up to 12 months after surgery. The fistula was considered as a new perianal fistula in case of occurrence at a distant localization. Recurrence was defined as reappearance of fistula in the same location within 12 months after initial healing. The time interval between surgery and recurrence was recorded.

### 2.7. Statistical Analysis

For statistical analysis, IBM SPSS Statistics 26.0 software (IBM, Armonk, New York, NY, USA) was used. Data was described by standard statistics, using median and range for continuous variables. All factors were analyzed for significant differences according to the Chi-square Pearson test. A *p*-value < 0.05 was considered significant.

## 3. Results

### 3.1. Study Population

The flowchart for inclusion is shown in Figure 1. From a total of 76 CD patients with perianal fistula, 31 patients were excluded in accordance with our exclusion criteria, leaving a cohort of 45 patients. Details are provided in Table 1. The patient characteristics table exhibits a preponderance of male patients (*n* = 28, 62.2%) with a BMI of 24 kg/m^2^. The first appearance of perianal fistula was diagnosed at a median age of 27 and the most frequent manifestations of CD outside the perianal area were localized in the ileocolon (L3, *n* = 18, 40%). The time interval from the initial diagnosis of CD until the first fistula surgery was 72 (0–391) months (Table 2).

### 3.2. Clinical Appearance of Perianal Fistulas

Two thirds of the cohort showed singular perianal fistulas (*n* = 29, 64.4%), in most cases accompanied by perianal abscesses (*n* = 26, 57.8%). The course of simple (*n* = 23, 51.1%) or complex (*n* = 22, 48.9%) fistulas was notably transsphincteric (*n* = 30, 66.7%) and located dorsal in the 4°°–8°° lithotomy position (*n* = 27, 60%) (Table 3).

### 3.3. Surgical Treatment

Details of surgical treatment are depicted in Table 4. In the 45 patients of the study, a total of 189 surgical procedures were performed: fistula seton drainage (*n* = 70), abscess incision (*n* = 50), fistulotomy (*n* = 34), AFP (*n* = 18), OTSC (*n* = 7), and mucosa advancement flap (*n* = 3). Forty patients (88.9%) needed a seton drainage as primary surgery before fistula closure surgery was performed. The seton was left in place for 5 (1–54) months until fistula closure (Table 2).

A wide variety of combinations of surgical techniques have been performed, which cannot all be presented here because of their diversity. Therefore, we chose to focus on the analysis of the very last surgical procedure performed before follow-up: in this regard, the three most common fistula therapies were fistulotomy (*n* = 14, 31.1%), removal of seton under biologicals (*n* = 12, 26.7%), and AFP (*n* = 8, 17.8%).

In 15 patients (33.3%), the first attempt to close the inner fistula ostium was sufficient to achieve healing. In 25 cases (55.6%), no closing procedure was applied and a fistulotomy (*n* = 10) or a fistula seton drainage (and its removal) (*n* = 11) were able to achieve healing.

A total of 4.2 (1–14) surgical interventions (including all abscess incisions, repeated fistula seton drainage, and fistula closing surgery) were performed per patient. The overall treatment period (from first to last surgery) was 18 months (0–128) (Table 2).

A diverting ileostomy was carried out in two patients with reversal after approximately 11 months.

### 3.4. Surgical Outcome

**Figure 2:**
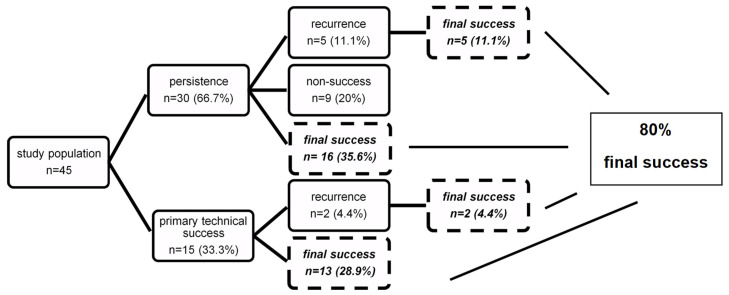
Flow diagram showing success of procedure.

Operative success is shown in Figure 2 whereas Table 5 presents detailed fistula characteristics of persistent, recurrent, and non-closing fistulas. The final success rate of fistula closure was 80%. The primary healing rate was 33.3%. Thirty patients (66.7%) experienced fistula persistence despite technical closure following initial surgery, as fistula secretion did not stop in time. Persistent fistulas were mostly complex with multiple external openings in dorsal localization.

Seven patients (15.5%) experienced a recurrence: two of them experienced a recurrence after an initial technical success five of them had initially persistent fistulas, then healed, then relapsed at a later stage. Recurrence of fistula occurred 45 (17–111) months after the last surgery (Table 2). Following 1.86 (1–2) surgical interventions (including all abscess incisions, repeated fistula seton drainage, and fistula closure surgery), all recurrent fistulas healed uneventfully, mostly following the application of an OTSC.

For nine patients (20%), among whom four declined further surgery and kept the seton in place as final therapy, overall treatment was finally not successful.

Three surgical complications were observed in two patients: one loss of AFP after two weeks and two OTSC dislocation occurrences. Neither postoperative abscesses nor fecal incontinence occurred.

### 3.5. Determinants for Successful Treatment, Persistence, and Recurrence

By univariate analysis, factors associated with successful primary treatment, persistence, and recurrence were identified (Table 6). A BMI of 18.5–24.9 kg/m^2^ (*p* = 0.020) and absence of leukocytosis (*p* = 0.040) were significantly associated with successful treatment of perianal fistulas. On the contrary, age 21–30 years at initial appearance of the fistula (*p* = 0.037) and low transsphincteric course (*p* = 0.032) correlated with fistula persistence. Regarding fistula recurrence, female sex (*p* = 0.046), complex fistulas (*p* = 0.034), high transsphincteric fistulas (*p* = 0.004), and fistulas in ventral localization (*p* = 0.020) were identified as relevant factors. Because of small numbers in all three groups, multivariate analysis failed to identify independent factors.

## 4. Discussion and Conclusions

This study analyzed 45 PFCD-patients over a period of 15 years with focus on outcome following minor, anal sphincter sparing surgery. We demonstrated a definitive closure rate of 80% in perianal fistulas for all individual patients with a median follow-up of 90 months. For a majority of patients, however, healing was achieved only after undergoing a combination of different surgical approaches, which had to be performed sequentially. In addition, we were able to identify factors influencing persistence, recurrence, and non-healing of perianal fistulas.

The demographics of our cohort were in line with other studies [1,3,13,14]. For example, almost 80% of the patients were aged under 40 years at initial fistula appearance, predominantly male and with normal weight. Main CD localization was ileocolonic. Cosnes et al. described that age under 40 corresponds with ano-perineal lesions with significant risk for penetrating complications in CD [15]. Other authors also described that two thirds of patients had ileocolonic or colonic CD [8]. Therefore, we believe that our cohort reflects a typical PFCD population.

In contrast to other series [8,14], our cohort presented similar numbers of simple and complex fistulas. More than 50% of patients suffered from perianal abscesses and, similarly to other studies, the most common first procedure to control local sepsis before fistula-closure-surgery was a seton drainage [14,16]. In our cohort, 4.2 surgical treatments were necessary (including all abscess incisions, repeated fistula seton drainage and fistula closure surgery) to achieve fistula healing, which took a median of 18 months. In contrast, comparable cohorts needed a median of six treatments for the closure of complex fistulas, and a median of three treatments for the closure of simple perianal fistulas [8]. However, the treatment of perianal fistulas in CD is very challenging and often patients require multiple treatments to achieve fistula healing. This has led to the development of new, innovative surgical procedures, such as AFP or OTSC [17,18], which have been applied in our cohort, too. Patients treated with mesenchymal stem cells or with fistula-tract laser closure (FiLaC) were excluded from our study, because these procedures were introduced in our PFCD center only last year, and thus do not allow for the necessary long-term follow-up.

Fistulotomy was performed 34 times in 45 patients. Williams et al. found that this kind of aggressive surgical treatment could be applied in low fistulas, which minimally involve the anal sphincter, to preserve sphincter function [19]. Results from other studies confirmed the good applicability of fistulotomy for low fistulas [14,20]. In our study, low transsphincteric fistulas carried a risk for fistula persistence, if not treated by fistulotomy—but possibly with greater risk of sphincter damage.

Especially with mucosal involvement (proctitis), CD has a negative impact on fistula closure after mucosa advancement flap and high recurrence rates are common [21,22]. In our cohort, this anal sphincter sparing surgery was only performed three times, in two patients successfully as last closure surgery. Fistula closure was still performed despite mild proctitis (*n* = 5). Here we observed one persistent, one recurrent, two unsuccessful and only one successful closure of perianal fistula.

A total of 28 PFCD-patients (62%) were treated with biologicals. Studies showed a successful treatment with infliximab combined with minor surgery, with rates up to 75% of fistula closing, but also with recurrence rates up to 40% [23,24,25]. In 10 patients the removal of seton under pursued biological therapy was successful as final treatment.

AFP combined with a mucosa advancement flap was implemented 18 times. In attention to the last closure surgery, AFP was performed eight times. According to literature, AFP was initially used frequently in PFCD surgery, after good results in small series [26,27]. Later AFP lost its importance due to its recurrence rate in systematic reviews [28] and especially due to its low (<35%) healing rates in a prospective study [29].

In four cases with recurrent complex fistulas, OTSC achieved a successful final fistula closure and thus proved to be an effective procedure [30]. Although proctectomy or final stoma deviation rates of up to 38% are described in other series with complex perianal fistulas, only 25% of our initial 76 PFCD patients received such a final therapy and only two patients needed a fistula surgery under fecal diversion with successfully final reversal without relapse [8,31,32,33]. This reflects the intention to avoid proctectomy or fecal diversion in young patients. In particular, an additional primary treatment option is to keep the “loose” seton as final therapy [34], which four patients favored in our series. Five other patients kept their seton drainage as permanent therapy after unsuccessful fistula treatment.

We differentiated between primary healing (33.3%), persistence despite technical closure (66.7%), and recurrence (15.5%). The relatively high rate of persistence is due to our definition of persistence and recurrence, but other series do not distinguish this aspect [14]. Regarding recurrence, it is difficult to compare our results with others (up to 60% in 48 months after surgery), because often cryptoglandular and CD perianal fistula are mixed, or only one or two surgical procedures are illuminated [14,20,35,36,37]. In our study recurrence occurred in seven patients, mostly with a high transsphincteric fistula and treated before recurrence by AFP, removal of seton under pursued biological therapy, or by fistulotomy. Other series have already identified complex perianal fistulas as a risk factor for recurrence [36]. We have found additional factors for persistence and recurrence, e.g., age, gender, course of perianal fistula. We are aware that some significant *p*-values include groups with patients <5 and in the cohort of 45 patients this is probably a type 2 error.

Resolving these limitations in our study will require further analysis: it is a retrospective study with a relatively small population, which makes it difficult to perform a reliable multivariate analysis with the purpose of recommending a standardized approach in PFCD surgery. Instead, patients are treated individually at the surgeon’s discretion [16]. Additionally, it is difficult to analyze the surgical procedures without bias in CD cohorts due to the wide variety of additive drug therapies, e.g., new biologicals with positive effect on PFCD [38,39,40]. Finally, different definitions of fistula healing exist in the literature. The clinical diagnosis of fistula healing is normally based on the symptomless closure of the internal and external opening. Some authors are of the opinion that no definitive statement can be made without a postoperative MRI. However, this approach can be questioned, as postoperative MRIs are usually not performed in a consistent manner to assess whether the fistula tract is really closed, simply behaving asymptomatically, or presenting side tracts that are still open and likely to cause fistula recurrence later [20,41,42]. Most studies lack a comparison between new surgical techniques [17]. Therefore, it seems too early to formulate recommendations about optimal surgical management strategies for the treatment of PFCD. Determining new surgical standards will require further larger, prospective, multicenter studies.

## Figures and Tables

**Table 1:** Patient characteristics.

		*n* (%) *
**age at initial fistula appearance**	*median*	27 (16–71) **
	<20	5 (11.1)
	21–30	23 (51.1)
	31–40	7 (15.6)
	41–50	4 (8.9)
	51–60	4 (8.9)
	>61	2 (4.4)
**sex**	female	17 (37.8)
	male	28 (62.2)
**BMI (kg/m^2^)**	*median*	24 (17.1–33.2) **
	<18.5	6 (13.3)
	18.5–24.9	24 (53.3)
	25–29.9	11 (24.5)
	30–34.9	4 (8.9)
	>35	0 (0)
**ASA**	ASA 1	13 (28.9)
	ASA 2	32 (71.1)
	>ASA 3	0 (0)
**smoking history**	yes no	8 (17.8) 37 (82.2)
**medication history**	steroids	13 (28.9)
	azathioprine	18 (40)
	biologicals	28 (62.2)
**surgical history**	abdominal IBD surgery	16 (35.6)
	proctosurgery	23 (51.1)
**Montreal classification for CD**	**age at diagnosis (A)**	
	A1, 16 years or younger	2 (4.4)
	A2, 17–40 years	35 (77.8)
	A3, >40 years	8 (17.8)
	**location (L)**	
	L1, terminal ileum	14 (31.1)
	L2, colon	11 (24.4)
	L3, ileocolon	18 (40.0)
	L4, upper GI	2 (4.4)
	**behavior (B)**	
	B1, non-stricturing/penetrating	0 (0)
	B2, stricturing	0 (0)
	B3, penetrating	0 (0)
	**perianal disease modifier (p)**	
	B3p, penetrating + perianal	45 (100)

* = study population *n* = 45; ** = median (range).

**Table 2:** Time intervals.

time interval	*n* (%) *	months ***
diagnosis of CD—first fistula surgery	27 (60) **	72 (0–391)
first—last fistula surgery	45 (100)	18 (0–182)
fistula seton drainage—fistula closure surgery	40 (88.9)	5 (1–54)
last surgery—last follow-up	45 (100)	90 (15–200)
last fistula closure surgery—surgery for recurrence	7 (15.5)	45 (17–111)

* = study population *n* = 45; ** = this parameter was only available in 27 patients; *** = median (range).

**Table 3:** Fistula characteristics.

		*n* (%) *
preoperative examination	perianal abscess	26 (57.8)
	anal fissure	5 (11.1)
	proctitis	5 (11.1)
	anorectal stricture	5 (11.1)
Park´s classification	superficial	4 (8.9)
	transsphincteric (low)	19 (42.2)
	transsphincteric (high)	11 (24.4)
	intersphincteric	3 (6.7)
	suprasphincteric	3 (6.7)
	extrasphincteric	5 (11.1)
AGA classification	complex	22 (48.9)
	simple	23 (51.1)
localization	ventral (10°°–2°°)	9 (20)
	dorsal (4°°–8°°)	27 (60)
	horizontal (9°° + 3°°)	9 (20)
number of fistulas	1	29 (64.4)
	≥2	16 (35.6)

* = study population *n* = 45.

**Table 4:** Surgical treatment details.

**surgical procedures in total**	abscess incision	50
	fistula seton drainage	77
	fistulotomy	34
	AFP	18
	OTSC	7
	mucosa advancement flap	3
		*n* (%) *
**surgery for infection control **	abscess incision	26 (57.8)
	fistula seton drainage	40 (88.9)
**last fistula closure surgery**	fistulotomy	14 (31.1)
	biologicals + seton removal	12 (26.7)
	AFP	8 (17.8)
	OTSC	5 (11.1)
	fistula seton drainage	4 (8.9)
	mucosa advancement flap	2 (4.4)
**number of attempts to close internal ostium**	0 (=fistulotomy, fistula seton drainage)	25 (55.6)
	1	15 (33.3)
	2	4 (8.9)
	3	1 (2.2)
**number of all surgical procedures**	*mean*	4.2 (1–14) **
**surgery under fecal diversion**		2 (4.4)
**PDAI before last fistula surgery**	*median*	8 (1–16) **

* = study population *n* = 45; ** = mean or median (range).

**Table 5:** Surgical outcome.

		*n* (%) *
**persistence**		30 (66.7) *
	low fistula	14 (46.7)
	high fistula	16 (53.3)
	complex	16 (53.3)
	ventral (10°°–2°°)	7 (56.7)
	dorsal (4°°–8°°)	16 (53.3)
	horizontal (9°° + 3°°)	7 (56.7)
	multiple fistulas	13 (43.3)
**recurrence**		7 (15.5) *
	low fistula	1 (14.3)
	high fistula	6 (85.7)
	complex	6 (85.7)
	ventral (10°°–2°°)	4 (57.1)
	dorsal (4°°–8°°)	3 (42.9)
	multiple fistulas	2 (28.6)
surgery before recurrence	biologicals + seton removal	3 (42.9)
	AFP	3 (42.9)
	fistulotomy	1 (14.2)
number of surgical procedures after recurrence	*mean*	1.86 (1–2) **
final fistula closure surgery after recurrence	OTSC	4 (57.1)
	fistulotomy	1 (14.3)
	mucosa advancement flap	1 (14.3)
	biologicals + seton removal	1 (14.3)
**non-success**		9 (20) *
	low fistula	6 (66.7)
	high fistula	3 (33.3)
	complex	3 (33.3)
	dorsal (4°°–8°°)	7 (77.8)
	horizontal (9°° + 3°°)	2 (22.2)
	multiple fistulas	4 (44.4)
last surgery before non-success	fistula seton drainage	4 (44.4)
	biologicals + seton removal	2 (22.2)
	fistulotomy	1 (11.1)
	AFP	1 (11.1)
	OTSC	1 (11.1)
postoperative complications		3 (6.7)
**final success**		36 (80) *
last surgery before success	fistulotomy	13 (28.9)
	biologicals + seton removal	10 (22.2)
	AFP	7 (15.7)
	OTSC	4 (8.9)
	mucosa advancement flap	2 (4.4)

* = study population *n* = 45; ** = mean (range).

**Table 6:** Significant factors in univariate analysis.

factor	persistence *n* (%) *		*p*-value	recurrence *n* (%) *		*p*-value	primary success *n* (%) *		*p*-value
	yes	no		yes	no		yes	no	
**sex** **female**	10 (58.8)	7 (41.2)	0.384	5 (29.4)	12 (70.6)	**0.046**	12 (70.6)	5 (29.4)	0.219
**male**	20 (71.4)	8 (28.6)		2 (7.1)	26 (92.9)		24 (85.7)	4 (14.3)	
**age at first appearance** **≤20**	1 (20)	4 (80)	**0.037**	0 (0)	5 (100)	0.812	5 (100)	0 (0)	0.197
**21–30**	14 (60.9)	9 (39.1)		5 (21.7)	18 (78.3)		20 (86.9)	3 (13.1)	
**31–40**	7 (100)	0 (0)		1 (14.3)	6 (85.7)		3 (42.9)	4 (57.1)	
**41–50**	3 (75)	1 (25)		1 (25)	3 (75)		3 (75)	1 (25)	
**51–60**	4 (100)	0 (0)		0 (0)	4 (100)		3 (75)	1 (25)	
**≥61**	1 (50)	1 (50)		0 (0)	2 (100)		2 (100)	0 (0)	
**BMI (kg/m^2^)** **≤18.5**	3 (50)	3 (50)	0.532	2 (33.3)	4 (66.7)	0.466	4 (66.7)	2 (33.3)	**0.020**
**18.5–24.9**	15 (62.5)	9 (37.5)		4 (16.7)	20 (83.3)		21 (87.5)	3 (12.5)	
**25–29.9**	9 (81.8)	2 (18.2)		1 (9.1)	10 (90.9)		10 (90.9)	1 (9.1)	
**30–34.9**	3 (75)	1 (25)		0 (0)	4 (100)		1 (25)	3 (75)	
**≥35**	0 (0)	0 (0)		0 (0)	0 (0)		0 (0)	0 (0)	
**Park’s classification** **superficial**	1 (25)	3 (75)	**0.032**	0 (0)	4 (100)	**0.004**	3 (75)	1 (25)	0.190
**low transsphincteric**	13 (68.4)	6 (31.6)		1 (5.3)	18 (94.7)		14 (73.7)	5 (26.3)	
**high transsphincteric**	9 (81.8)	2 (18.2)		6 (54.5)	5 (45.5)		10 (90.9)	1 (9.1)	
**intersphincteric**	0 (0)	3 (100)		0 (0)	3 (100)		3 (100)	0 (0)	
**suprasphincteric**	3 (100)	0 (0)		0 (0)	3 (100)		1 (33.3)	2 (66.7)	
**extrasphincteric**	4 (80)	1 (20)		0 (0)	5 (100)		5 (100)	0 (0)	
**AGA classification** **simple**	14 (60.9)	9 (39.1)	0.399	1 (4.3)	22 (95.7)	**0.034**	17 (73.9)	6 (26.1)	0.297
**complex**	16 (72.7)	6 (27.3)		6 (27.3)	16 (72.7)		19 (86.4)	3 (13.6)	
**localization of fistula** **ventral (10°°–2°°)**	7 (77.8)	2 (22.2)	0.435	4 (44.4)	5 (55.6)	**0.020**	9 (100)	0 (0)	0.238
**dorsal (4°°–8°°)**	16 (59.3)	11 (40.7)		3 (11.1)	24 (88.9)		20 (74.1)	7 (25.9)	
**horizontal (9°° + 3°°)**	7 (77.8)	2 (22.2)		0 (0)	9 (100)		7 (77.8)	2 (22.2)	
**leukocytes at last fistula closure surgery** **≤12.680/μL** **≥12.680/μL**	23 (63.9) 7 (78.2)	13 (36.1) 2 (22.2)	0.429	5 (13.9) 2 (22.2)	31 (86.1) 7 (77.8)	0.537	31 (86.1) 5 (55.6)	5 (13.9) 4 (44.4)	**0.040**

Bold values indicate significance (*p*-value ≤ 0.05, long rank test). * = study population *n* = 45.

## Data Availability

All available data is contained in this article.
